# Activity of pontine A7 noradrenergic neurons is suppressed during REM sleep

**DOI:** 10.1152/japplphysiol.00771.2021

**Published:** 2022-05-26

**Authors:** Victor B. Fenik, Irma Rukhadze

**Affiliations:** ^1^Department of Neuroscience and Experimental Therapeutics, Albany Medical College, Albany, New York; ^2^VA Greater Los Angeles Healthcare System, West Los Angeles, California; ^3^Department of Medicine, David Geffen School of Medicine at UCLA, Los Angeles, California

**Keywords:** head-restrained rat, hypoglossal motoneurons, norepinephrine, REM sleep, sleep apnea

## Abstract

The activity of hypoglossal motoneurons plays an important role in the maintenance of upper airway patency. Both withdrawal of noradrenergic excitatory drive and increase of cholinergic inhibition markedly decrease excitability of hypoglossal motoneurons during sleep and especially during rapid-eye-movement (REM) stage. This leads to increased collapsibility of upper airway during sleep, which is the major neurological factor of obstructive sleep apnea (OSA) pathophysiology. Anatomical and functional data suggest that noradrenergic A7 neurons are the main source of noradrenergic drive to hypoglossal motoneurons. However, it is unknown whether the behavior of A7 neurons during sleep-wake cycle is in accord with their proposed involvement in sleep-related depression of hypoglossal motoneuron activity. Therefore, we sought to assess the behavior of A7 neurons during sleep and wakefulness in naturally sleeping head-restrained rats. We have found that, similar to other pontine noradrenergic neurons, the putative A7 noradrenergic neurons fired with relatively long-lasting action potentials with a low-frequency regular discharge. Importantly, noradrenergic A7 neurons were predominantly silent during REM sleep. The REM-off activity of the A7 neurons supports our hypothesis that these neurons may significantly contribute to the withdrawal of excitatory noradrenergic drive from upper airway motoneurons during REM sleep and, consequently, play an essential role in maintaining upper airway patency and pathophysiology of OSA. Therefore, noradrenergic A7 neurons may serve as an additional target for designing pharmacological approaches to treat OSA.

**NEW & NOTEWORTHY** Noradrenergic A7 neurons are mostly silent during REM sleep. This is in accord with their role in the control of upper airway muscles and important contribution to OSA pathophysiology. Therefore, a modulation of A7 neuron activity can serve as a novel therapeutic target for pharmacological treatment of OSA.

## Graphical abstract

**Figure:**
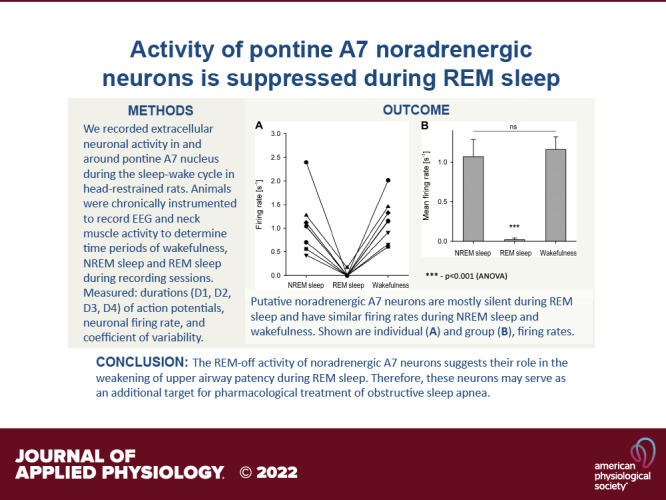

## INTRODUCTION

Patients with obstructive sleep apnea (OSA) have collapsible upper airway (UA) (1–4). To compensate for the insufficient UA patency, the activity of genioglossus muscle, which is innervated by hypoglossal motoneurons, is elevated during wakefulness in patients with OSA (5). However, during sleep and especially during rapid-eye-movement (REM) sleep, the compensatory hypoglossal motoneuron activity is markedly reduced. This phenomenon leads to partial or complete closure of UA causing repeated nocturnal hypopnea/apnea events in patients with OSA (4–7). The brainstem neuronal circuitry that is responsible for depression of hypoglossal motoneuron activity during non-REM (NREM) and REM sleep as well as neurochemical mechanisms that contribute to this depression are not completely understood (8–16). Two neurochemical mechanisms, the withdrawal of noradrenergic drive from and postsynaptic cholinergic inhibition of hypoglossal motoneurons, have received the most experimental support as key mechanisms of REM sleep-related depression of hypoglossal motoneurons (17–21).

Neuroanatomical studies have determined that the major noradrenergic afferent projections to the hypoglossal nucleus originate from brainstem catecholaminergic nuclei of A1/C1, A5, A7, and SubCoeruleus (SubC) (22–25). In functional studies, suppression of pontine noradrenergic neurons by local application of clonidine, which is an α2-adrenergic agonist that activates inhibitory autoreceptors located on noradrenergic neurons, revealed that inhibition of A7 neurons effectively decreased spontaneous activity in hypoglossal nerve (26). In contrast, the clonidine-induced inhibition of either A5 or SubC neurons failed to affect hypoglossal nerve activity in the same animal preparations (26–28). Thus, A7 neurons have been demonstrated to provide the major state-dependent noradrenergic drive to hypoglossal motoneurons (26).

The noradrenergic locus coeruleus (LC) and SubC neurons reduce their firing rate during NREM sleep and become silent during REM sleep in naturally sleeping animals (29, 30). Also, noradrenergic LC and A5 cells have been shown to be silent during carbachol-induced REM sleep-like state in anaesthetized rats (27, 31). Noradrenergic A7 neurons have been suggested to have a state-dependent activity based on cFos studies in behaving and anesthetized rats (32, 33). However, the discharge pattern of A7 neurons during natural sleep-wake cycles has not been directly investigated. Thus, the goal of this study was to determine the activity of A7 neurons during natural sleep and wakefulness to corroborate their role in the mechanisms of depression of hypoglossal motoneuron activity during both NREM sleep and REM sleep.

We have found that all, but one, recorded putative noradrenergic A7 neurons were silent during REM sleep. A preliminary report has been published (34).

## METHODS

The reported data are from 5 adult male Sprague-Dawley rats (body weight: 360–400 g; Charles River Laboratories). The animals were housed in the Greater Los Angeles VA Healthcare System vivarium under a 12/12-h light/dark cycle (light was on at 6 AM and off at 6 PM) with standard rodent food and water available ad libitum. All experimental procedures were approved by the Institutional Animal Care and Use Committee of the Greater Los Angeles VA Healthcare System and were conducted in accordance with the National Institutes of Health Guide for the Care and Use of Laboratory Animals.

### Surgical Implantation of Electrodes

Animals were initially anesthetized with isoflurane (2%) followed by a mixture of ketamine/xylazine (80/7.5 mg/kg im, supplemented with 30 mg/kg ketamine, as needed). The animals were premedicated with an analgesic buprenorphine HCl (0.05 mg/kg sc; Hospira, Inc., Lake Forest, IL) and an antibiotic Baytril (5 mg/kg sc; Bayer HealthCare LLC, Shawnee Mission, KS). The rats were placed in a stereotaxic instrument (David Kopf Instruments, Tujunga, CA) in the prone position and the animal’s head positioned at 15° so that the bregma level was lower than the lambda level. The animals were implanted for recording of the electroencephalogram (EEG) and bilateral neck trapezius muscle electromyogram (EMG), activity of which is commonly used to monitor postural muscle tone during sleep-wake cycles. For recording EEG, two screws were positioned in the skull: one in the frontal bone (2-mm rostral and 2-mm lateral to the bregma) and one in the parietal bone contralaterally (3-mm caudal and 2-mm lateral to bregma). The screws were attached to Teflon-coated stainless steel wires (0.002/0.0045 in. bare/coated diameters, A-M Systems, Carlsborg, WA). Additional screws were placed bilaterally in the parietal bone to strengthen the attachment of acrylic to the skull. Neck EMG was recorded with electrodes (0.002/0.0045 in. bare/coated diameters, A-M Systems, Carlsborg, WA) placed bilaterally in the dorsal neck musculature. The wires from the EEG and EMG electrodes were soldered to male miniature pin connectors (A-M Systems), which were affixed to the calvarium with acrylic cement (A-M Systems). An opening was made in the parietal bone at the midline caudal to the lambda to permit the subsequent insertion of glass electrodes for recording of neuronal activity during states of sleep and wakefulness. Between experimental sessions, this opening in the skull was closed using a stainless screw to cover and protect the brain surface from damage and infections. Acrylic cement was used to build the acrylic head-plug, which contained four laterally directed openings (two on each side), which were used to restrain the animal’s head during experimental sessions.

### Postoperative and Habituation Procedures

Postoperative care included twice-a-day observation of the animal’s behavior and injections of buprenorphine and Baytril to alleviate possible postsurgical discomfort and to prevent infection, respectively. During both habituation and experimental sessions, rats were placed in up to animal size elongated boxes to limit their body movements. Initially, each rat, implanted with EEG and EMG electrodes for sleep-wake recordings, was habituated to the box without the head restraint and, subsequently, the habituation was performed under the head-restrained condition (semi-chronic headholder-880; David Kopf Instruments). To minimize the discomfort of the animals, the duration of the habituation sessions increased gradually over several days depending on the individual rat’s behavior under the head-restrained condition. Following an adaptation period of 4–6 days, the animals did not exhibit any sign of distress when placed in head-restraining device and fell asleep within minutes. During recording sessions of 4–5 h, they exhibited normal transitions between wakefulness, NREM sleep, and REM sleep (35).

### Experimental Procedures

During experimental sessions, the animal’s head was immobilized in the head-restraining device and the male connectors in the head-plug were attached to the recording equipment to monitor the EEG and neck EMG. The protective screw was removed under aseptic conditions with local anesthesia (Bupivacaine HCl) to alleviate possible animal’s discomfort during the insertion of recording electrodes. For single-unit recordings, glass electrodes were positioned stereotaxically at angles in both rostral (0–8°) and lateral (14–20°) directions. Using a hydraulic micropositioner (model 650, David Kopf Instruments), the electrodes were advanced to the depth of 5–8 mm to record extracellular activity of noradrenergic A7 neurons and other neurons located in or around to A7 nucleus. At the end of each experimental session, the opening in the skull was closed with a protective screw before returning the rat to its home cage.

### Signal Recording and Measurements

We recorded extracellular unit activity within and nearby the A7 nuclei, which are bilaterally located in the dorsolateral pons just under the Kolliker-Fuse nuclei at the following stereotaxis coordinates: 8.72–8.80 caudal to bregma, 2.8 mm lateral to midline, and interaural 1.8 mm. For extracellular recording of neuronal activity, glass electrodes (A-M Systems) were pulled using a vertical pipette puller (Model 720, David Kopf Instruments) and their tips were broken under microscope to 2–4 µm. The electrodes were filled with 0.5 M sodium acetate that contained 2% Pontamine sky blue (Sigma-Aldrich, St. Louis, MO) to iontophoretically mark the recording site. The activity of noradrenergic A7 neurons was recorded within a localized region of the dorsolateral pontine tegmentum with the following recording coordinates: rostral angles 2–7.5°, lateral angles 15.5–16.7°, and depths 7–8 mm, from the midline of cerebellum surface. We recorded each animal for several recording sessions (up to 10 days), during which we used new electrode tracks to search for active neurons in the lateral pontine area.

EEG and neck EMG signals were amplified (Differential AC Amplifier 1700; A-M Systems) and filtered with a bandwidth of 0.1–500 Hz and 10–5 kHz, respectively. Extracellular neuronal activity was recorded by an AxoClamp 2B amplifier (Axon Instruments, Inc., Union City, CA) and conditioned with a differential amplifier (Model DP-304, Warner Instruments, LLC, Hamden, CT) that had a filter bandwidth of 0.3–3 kHz. During experiments, all signals were monitored with oscilloscopes, digitized by A/D converter Digidata 1322 A or Power-1401 A/D converters, and saved in a computer using AxoGraph software (Axon Instruments) or Spike-2 v.7 data acquisition system (Cambridge Electronic Design, Inc., Cambridge, UK), respectfully, at 20 kHz sampling rate.

All data reported in this study were collected from 12 PM to 5 PM. The sleep and wakefulness states were identified using EEG power spectrum, which clearly showed theta rhythm during REM sleep and visual analyses of both EEG and neck EMG signals as described previously (35). Using averaged signal of action potentials, we calculated the action potential distances D1, D2, D3, and D4, as described in Ref. 36. Coefficient of variability (CV) was calculated for each state as standard deviation/mean interval of discharge and was used to classify neuronal discharges as regular (CV ≤ 0.5) and irregular (CV > 0.5). We also calculated the mean firing rate of recorded neurons in each state and used it as a criterion to judge if the neuronal discharge changed relative to the previous state. We have found that a difference in the mean frequency of 50% or more (50+%) was a good estimate of the state-dependent change of a neuron firing rate.

### Classification of Recorded Neurons

The neurons were classified based on changes in their average firing rate during wakefulness, NREM sleep, and REM sleep. “REM-active” neurons had similar firing rate (difference less than 50%) during NREM sleep and wakefulness but increased their activity by 50+% during REM sleep. “REM-OFF” neurons were silent or almost silent during REM sleep. “REM/wake-active” neurons had similar firing rates during REM sleep and wakefulness, which were higher by 50+% during NREM sleep. “NREM-REM-wake-gradient-up” (“NRW-gradient-up”) neurons had firing rate higher by 50+% during REM sleep than NREM sleep and by 50+% during wakefulness than REM sleep. “NRW-gradient-down” neurons had firing rate lower by 50+% during REM sleep than NREM sleep and lower by 50+% during wakefulness than REM sleep. In “state-independent” neurons, changes in the mean firing rate between the behavioral states remained within the 50% threshold.

### Histological Procedures

At the end of each series of the daily recording sessions, the rats were deeply anesthetized with isoflurane (5%) followed by ketamine/xylazine (100/10 mg/kg) and perfused transcardially with 500 mL of phosphate buffer saline (PBS, pH 7.4) at room temperature followed by 500 mL of 10% formalin in PBS. Brains were removed and postfixed overnight in formalin-PBS at 4°C. Following cryoprotection in 30% sucrose-PBS, brains were cut into 40-µm coronal sections using a cryostat.

The sections were washed in phosphate-buffered saline (PBS) and incubated with affinity-purified polyclonal antibody raised in rabbit against denatured tyrosine hydroxylase (TH, a marker for catecholaminergic neurons) from rat pheochromocytoma (EMD Millipore, Cat. No. AB152, RRID:AB_390204). Sections were incubated overnight at room temperature in the primary TH antisera diluted 1:2,000 in PBS that contained 0.25% Triton X-100 (PBST). After subsequent washing in PBS, they were incubated in biotinylated donkey anti-rabbit secondary antisera (Jackson Immunoresearch Labs Cat. No. 711-065-152, RRID:AB_2340593) diluted at 1:500 in the PBST solution for 2 h followed by the avidin-biotin peroxidase complex (Vector Laboratories, Vectastain Elite ABC Kit, Cat. No. PK-6100) treatment. Nickel-enhanced DAB with H_2_O_2_ reaction (Vector Laboratories, DAB Substtrate Kit, Peroxidase, with Nickel, Cat. No. SK-4100) that produced black staining was used to label pontine noradrenergic neurons. Sections were then washed in PBS, mounted on Superfrost Plus slides (Fisher Scientific, Cat. No. 12-550-15), dried overnight, stained with neutral red, and cover-slipped using the Permaslip mounting medium (Fisher Scientific, Cat. No. NC0530667). To verify the locations of recording sites in relation to A7 noradrenergic neurons, the sections were analyzed and digitized using the bright-field microscope (Leica DM2500).

The rabbit anti-TH antibody was used for the detection of A7 noradrenergic neurons. This antibody stains a single band of 62 kDa on Western blot corresponding to TH molecular weight (manufacturer’s technical information). The specificity of immunostaining for TH was indicated by the lack of detectable immunostaining in brain regions outside established groups of catecholamine neurons. The cellular morphology of TH-immunopositive (TH+) neurons and their distribution in A1/C1, A2/C2, A5, A6, A7, and SubC catecholaminergic regions in the rat brainstem sections stained with this antibody was essentially identical to that observed in previous studies (26, 32, 37). For the secondary antibody immunohistochemical control, the primary antibody was omitted that resulted in no immunoreactivity above the background.

### Statistical Analysis

The two-tailed *t* test, two-way ANOVA, and one-way repeated-measures (RM) ANOVA were used for statistical analyses of normally distributed data (SigmaPlot, Systat Software, Inc.). The main effect of RM ANOVA was the effect of state on neuronal firing rate. The Mann–Whitney and Holm–Sidak tests were applied for data that did not pass a normality test. The linear regression analysis was used to assess correlation between D3 and D2 durations of the action potential. The null hypothesis was rejected at the level of *P* < 0.05 and the standard error (SE) was used to describe the variability of the mean values.

## RESULTS

**Figure 1.**
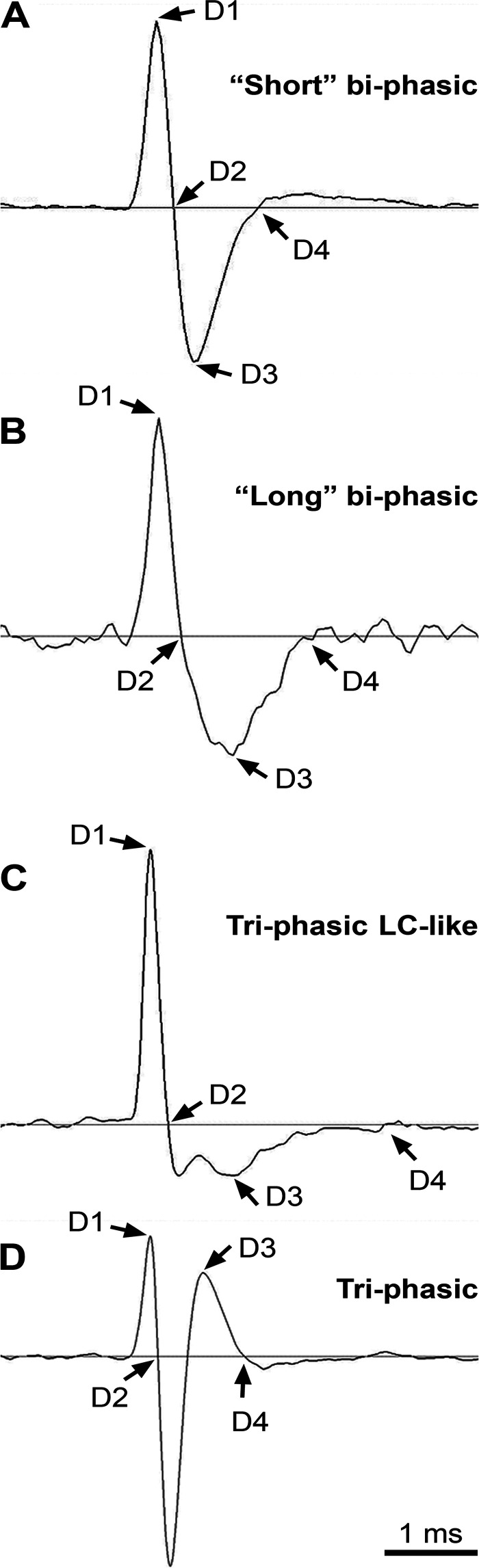
Shapes of action potentials (APs) of the recorded neurons. Each panel shows how AP durations D1, D2, D3, and D4 were measured using the average traces of extracellularly recorded APs. *A*: the “short” biphasic AP. *B*: the “long” biphasic AP. *C*: the “LC-like” AP with a “notch” occurring after the spike characteristic for LC cells. *D*: the triphasic action potentials. LC, locus coeruleus.

The main objective of this study was to determine the activity pattern of pontine noradrenergic neurons that are located in the A7 nucleus during sleep and wakefulness. During recording sessions, we also recorded spontaneously active units to be able to differentiate A7 neurons from other neurons by the shape and timings of action potentials (APs), their firing rate, CV, and pattern of activity. Most of the recorded neurons (72%) had “short” biphasic APs (Fig. 1*A*), whereas the remaining 28% of neurons had relatively “long” and more complex APs (Fig. 1, *B*–*D*). The mean durations of D1, D2, D3, and D4 for the “short” and “long” APs are reported in Table 1.

**Table 1.** Average durations for the “short” and “long” action potentials

	D1	D2	D3	D4
“Short” APs	0.27 ± 0.015	0.464 ± 0.029	0.676 ± 0.044	1.71 ± 0.15
“Long” APs	0.23 ± 0.02	0.43 ± 0.037	0.978 ± 0.056***	2.15 ± 0.21

Values are means ± SE, in seconds. A comparison between 33 “short” and 13 “long” action potentials (APs) by Mann–Whitney Rank Sum test. ***P < 0.001

**Figure 2.**
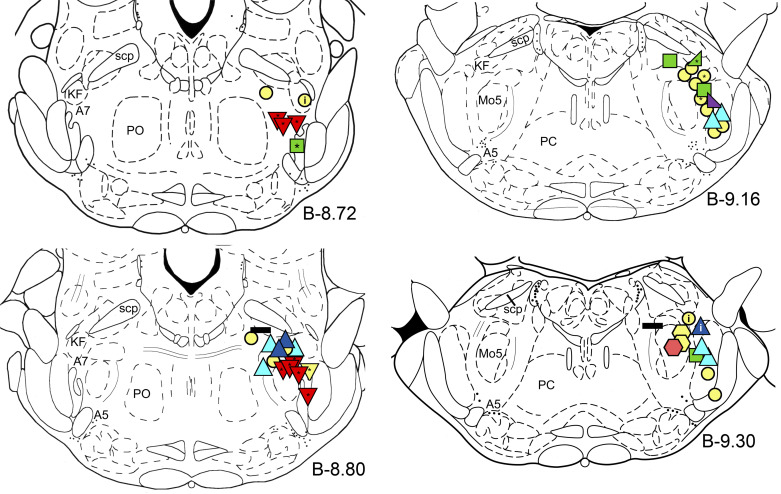
Distribution of the recording sites of all recorded neurons in this study, which are shown on pontine coronal sections at anteroposterior levels from bregma B-8.72 to B-9.30 (38). Blue triangles indicate “REM-active” neurons with low (blue) and high (dark blue) frequency discharges; red and yellow reverse triangles, “REM-OFF” putative noradrenergic A7 neurons; pink and yellow hexagons, putative trigeminal motoneurons; green squares, “REM/wake-active” neurons; green “rising” right triangle, “NRW-gradient up” neuron, purple “decreasing” right triangle, “NRW-gradient down” neuron; and horizontal black rectangles show “state-independent” neurons. Symbols colored in yellow indicate neurons, which activity was recorded only during one or two states and, therefore, their state-dependent behavior could not be determined. The asterisks inside the symbols show neurons with the “long” APs. The letter “i” inside the symbols indicates neurons whose activity was inspiratory modulated. AP, action potential; A5 and A7, noradrenergic A5 and A7 nuclei, respectively; KF, Kolliker-Fuse nucleus; Mo5, trigeminal motor nucleus; NRW-gradient up, NREM-REM-wake (NRW)-gradient up; PC, caudal pontine reticular nucleus; PO, oral pontine reticular nucleus; REM, rapid-eye-movement; scp, superior cerebellar peduncle.

The behavior of recorded cells was classified based on the changes of their firing rate across the sleep-wake cycles, which showed a wide spectrum of behavior patterns. The distribution of the recording sites of recorded neurons and their classification is shown in Fig. 2. From the total of 46 recorded neurons, the activity of 27 was observed during all three consecutive states (wakefulness, NREM sleep, and REM sleep). The recorded neurons were classified as “REM-active” neurons (blue and dark blue triangles), “REM-OFF” putative noradrenergic A7 neurons (red reversed triangles), putative trigeminal motoneurons (pink hexagon), “REM/wake-active” neurons (green squares), “NRW-gradient up” neurons (rising right triangle), “NRW-gradient down” neurons (decreasing right triangle), and “state-independent” neurons (horizontal black rectangles). The activity of the remaining neurons was recorded only during one or two states and, therefore, their state-dependent behavior could not be determined. The asterisks inside the symbols indicate neurons with the “long” AP. The letter “i” indicates that the activity of the recorded cell was inspiratory modulated (based on phasic excitation that coincided with visually observed movements of animal chest during each inspiratory effort).

**Figure 3.**
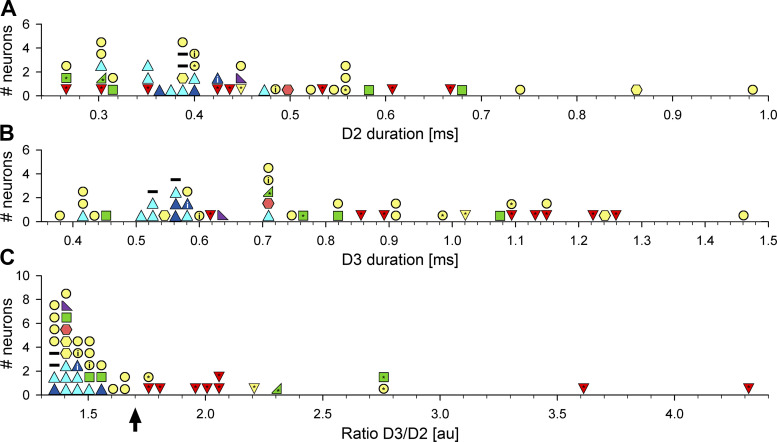
Histograms of AP durations D2 (*A*), D3 (*B*), and the ratio D3/D2. The meaning of symbols is the same as in Fig. 2. The arrow in *C* indicates the ratio 1.7 that separates neurons with the “short” and “long” action potentials. AP, action potential.

Even though the duration D3 was significantly different in the cells that have the “long” AP compared with the “short” AP cells (see Table 1), histograms of the distribution of either D2 or D3 durations did not separate between these neurons (Fig. 3, *A*and *B*). However, a histogram of the ratio of D3/D2 durations divided the “long” and “short” AP neurons at the ratio value 1.7 (Fig. 3*C*, arrow).

**Figure 4.**
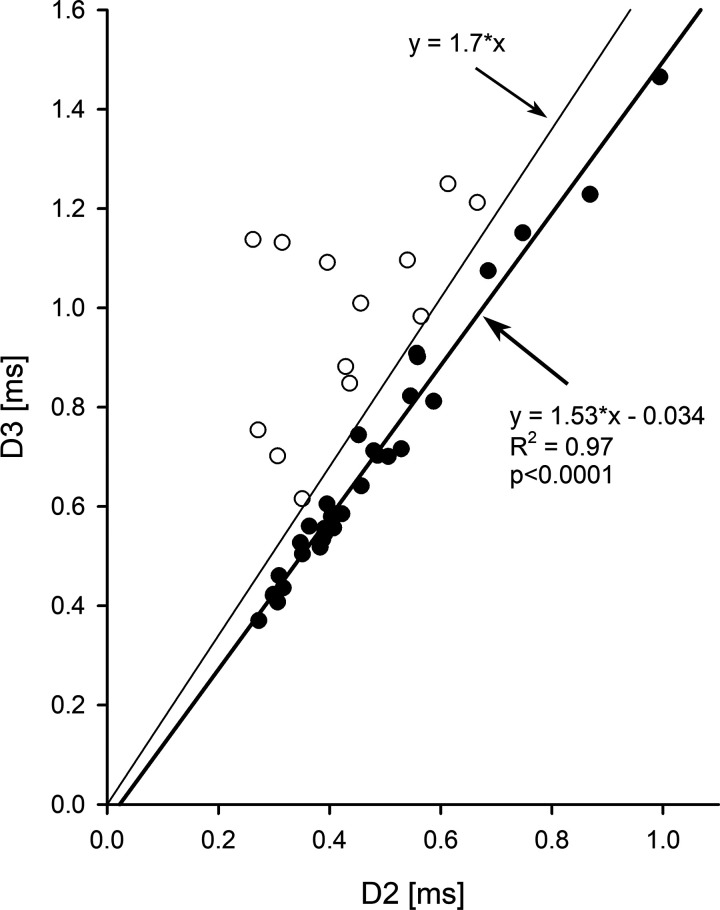
The plot of D3 vs. D2 action potential durations of each recorded neurons. Black and open circles correspond to neurons with the “short” and “long” action potentials, respectively. The linear regression analysis was done only for neurons with “short” action potentials (thick line), which shows a strong correlation between the D3 and D2. The plot of D3 = 1.7*D2 (thin line) separates the recorded neurons that discharged with the “short” and “long” action potentials.

In addition, there was a strong correlation between AP durations D2 and D3 for the neurons that have “short” biphasic APs (*R^2^* = 0.97, *P* < 0.0001, Fig. 4, a thick line). The symbols for the “long” AP cells were more widely distributed on the left side of the plot and the correlation between D2 and D3 was much weaker for those neurons (*R^2^* = 0.28, *P* = 0.06, regression line is not shown). In addition, a line with the slope 1.7 satisfactorily separated the symbols for neurons with the “short” and “long” APs (Fig. 4, a thin line). This suggests that the D3/D2 AP ratio can be beneficial for the classification of extracellularly recorded neurons. We also used two-way ANOVA test to verify if there is an effect of animal on the D3/D2 ratio. The test showed that the D3/D2 ratio of the neuron’s AP was significantly related to the neuron type (*F*_4,11 _ = _ _2.17; *P* < 0.05) and that there was no effect of animal, in which the cells were recorded (*F*_4,11_ = 0.66; *P* = 0.6).

### REM-Off Activity of Putative Noradrenergic A7 Neurons during Sleep and Wakefulness

**Figure 5.**
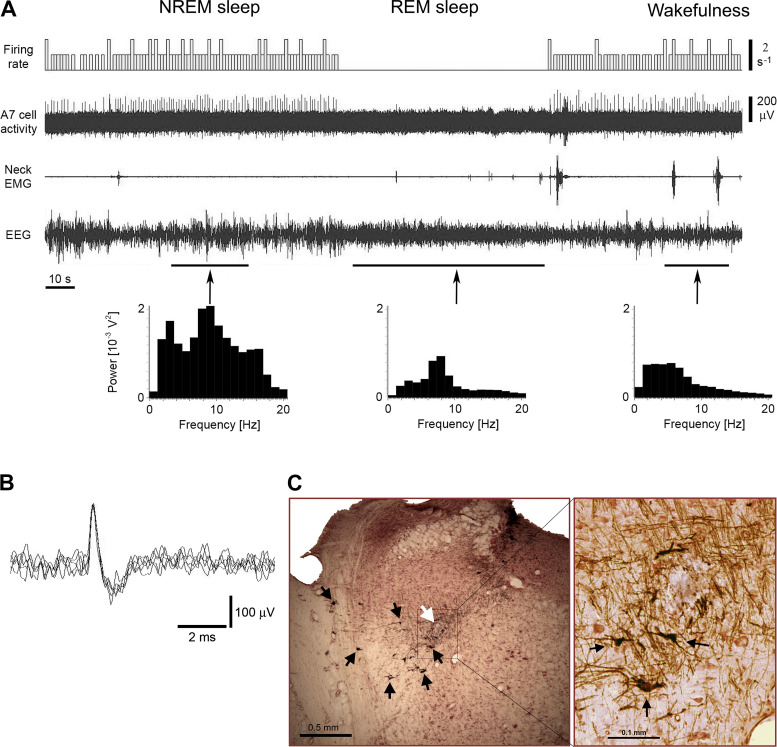
Extracellular recording of the activity of a putative noradrenergic A7 neuron. *A*: the A7 neuron discharged regularly with typically low firing rate during NREM sleep (average firing rate 1.04 s^−1^, CV 0.426) and wakefulness (average firing rate 1.16 s^−1^, CV 0.17), and was silent during REM sleep. The trace “Firing rate” shows bars that reflect the number of spikes within equal time intervals (in this case, 1 s) that are not synchronized with the state of animal. Power spectra of EEG that were calculated during time periods marked by arrows helped to properly detect behavioral states of the animal. Note the appearance of the theta rhythm in the EEG power spectrum during REM sleep in 7–8 Hz bands. *B*: superimposed five traces of the A7 neuron action potentials. *C*: a coronal pontine section stained by immunohistochemistry for TH and neutral red shows the recording site of this A7 neuron (white arrow) within the “loose” part of the A7 nucleus. Black arrows point to some TH-positive noradrenergic A7 neurons with their dendrites within the nucleus. The site was marked by iontophoretically applied Pontamine sky blue from the recording electrode after the end of recording. The black box indicates an area that is expanded and shown to the right. The expanded image shows additional TH-positive A7 neurons nearby the recording site. CV, coefficient of variability; NREM, non-rapid-eye-movement; REM, rapid-eye-movement; TH, tyrosine hydroxylase.

Nine putative noradrenergic A7 neurons were recorded within histologically identified A7 nuclei that was confirmed by the distribution of TH-positive A7 neurons (see Fig. 2, B-8.8 and B-8.72, inverted triangles). From the nine recorded A7 neurons, eight were recorded during all three behavioral states. These A7 neurons had the “long” type of APs (mean values: D1 = 0.244 ± 0.03 ms, D2 = 0.452 ± 0.051 ms, D3 = 1.02 ± 0.077 ms, D4 = 2.17 ± 0.24 ms; *n* = 8). Figure 5 shows the behavior of a representative A7 neuron that was recorded within the “loose” part of the A7 nucleus. In the “loose” part, noradrenergic neurons are localized with less density as compared with more rostral “compact” part of the A7 nucleus (26). The activity of A7 neurons was characterized by tonic pattern of activity with a relatively low firing rate during both NREM sleep (mean frequency: 1.07 ± 0.22 s^−1^, range: 0.43–2.39 s^−1^; mean CV: 0.399 ± 0.05, range: 0.28–0.729; *n* = 8) and wakefulness (mean frequency: 1.16 ± 0.16 s^−1^, range: 0.606–2.01 s^−1^; mean CV: 0.229 ± 0.025, range: 0.1–0.31; *n* = 8), which is typical for other pontine noradrenergic neurons.

**Figure 6.**
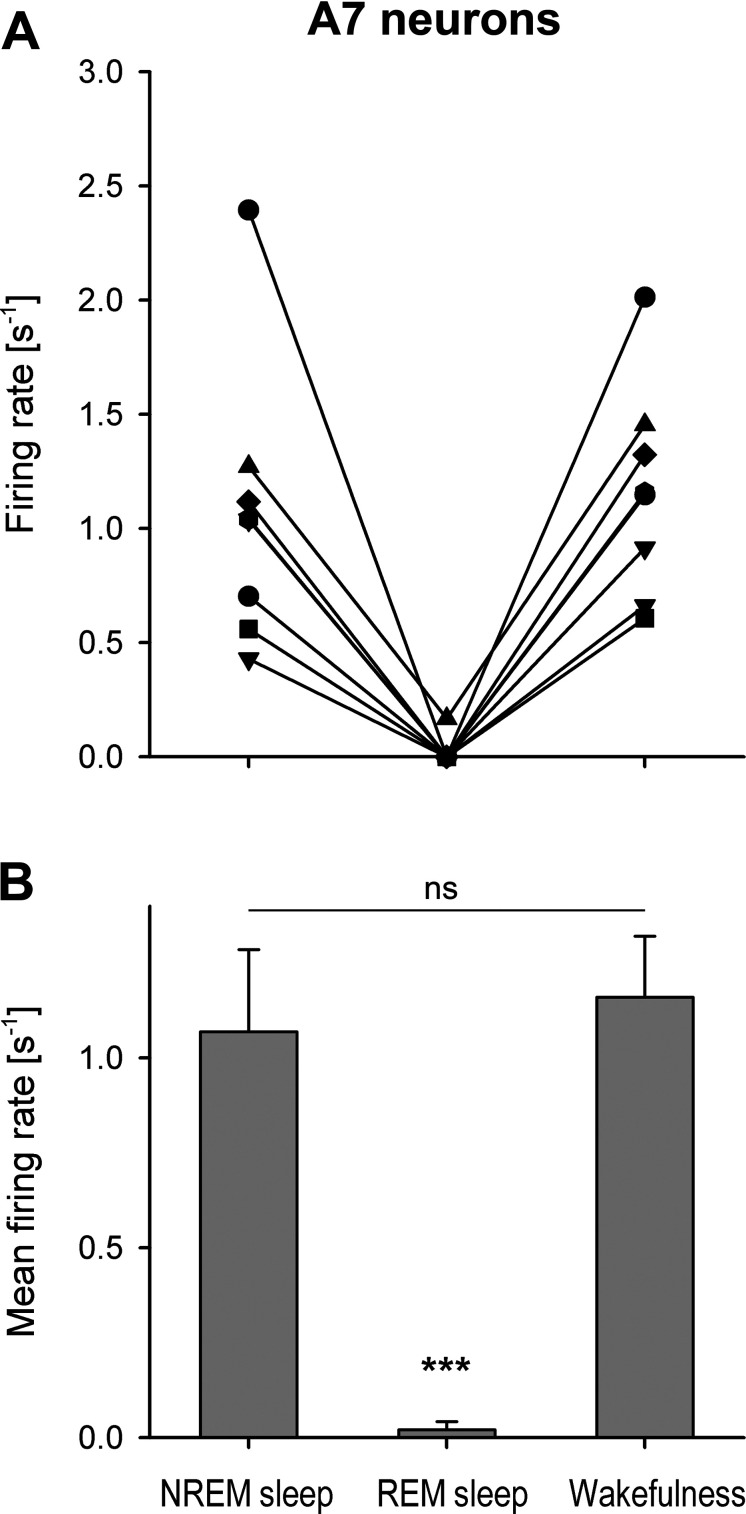
Individual (*A*) and group (*B*) firing rates of putative noradrenergic A7 neurons during sleep and wakefulness. *A*: individual firing rates of A7 cells are represented by unique symbols during the successive states of NREM sleep, REM sleep, and wakefulness. Note that all, but one, A7 cells were silenced during REM sleep. *B*: during REM sleep, the average firing rate of the A7 neurons were minimal and significantly different from that during NREM and wakefulness (***P < 0.001, one-way RM ANOVA). However, the mean firing rates of A7 cells were not different (*P* = 0.58) during NREM and wakefulness. NREM, non-rapid-eye-movement; REM, rapid-eye-movement.

During REM sleep, the recorded A7 neurons became silent (*n* = 7) or generated few spikes during this state (*n* = 1). On average, the putative A7 neurons had similar firing rates during NREM sleep and wakefulness (Fig. 6). The decrease in their activity during REM sleep was significant as compared with NREM sleep and wakefulness (*F*_2,7 = _30.7, *P* < 0.001, one way RM ANOVA, Fig. 6*B*). However, there was no statistical difference between firing rates of A7 neurons during NREM sleep and wakefulness (*P* = 0.58, pairwise comparison by Holm–Sidak method). The activity of one putative A7 neuron was recorded within the A7 nucleus only during wakefulness (Fig. 2, B-8.80, yellow reverse triangle). The parameters of its activity were similar to the other A7 cells that were recorded in this study (Fig. 2, red reverse triangles): AP durations were D1 = 0.242 ms, D2 = 0.456 ms, D3 = 1.0 ms, and D4 = 3.47 ms. The firing rate of this neuron was 1.02 s^−1^ (CV = 1.06) during wakefulness. In addition, the activity of all recorded A7 neurons was not sensitive to animal movements during grooming.

The waveform of AP was different among the nine recorded A7 neurons. Five neurons had a “long” biphasic shape of AP (see Fig. 1*B*), two neurons had an “LC-like” AP, i.e., triphasic shape with a “notch” that follows the spike (see Fig. 1*C*), and remaining two neurons had a triphasic AP shape (see Fig. 1*D*). These neurons were recorded in both the “loose” and “compact” parts of the A7 nucleus (26). Three neurons were recorded in the more rostral “compact” part (see Fig. 2, B-8.72, three red triangles) and the remaining six, in the caudal “loose” part of the nucleus (Fig. 2, B-8.80, five red and one yellow triangle). There was no relationship between the AP shape of these neurons and their location within the “loose” or “compact” parts of the A7 nucleus.

### REM-Active Neurons

**Figure 7.**
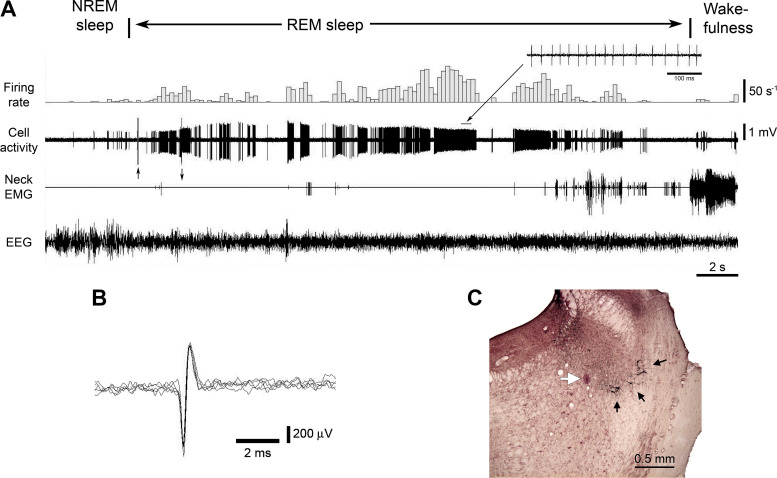
Example of the activity of a REM-ON neuron during sleep and wakefulness. *A*: the neuron had an irregular discharge that dramatically increased during REM sleep (average firing rate 19.7 s^−1^, CV 4.44) as compared with NREM sleep (average firing rate 0.942 s^−1^, CV 1.63) and wakefulness (average firing rate 2.33 s^−1^, CV 2.92) (see also *inset* showing cell discharge at near maximal frequency). Note the changes in the amplitude of action potentials, which were likely due to brain micro-movements relative to the tip of recording electrode that occurred during transition between the behavioral states. The arrows pointed up and down at the beginning of the record indicate artifacts that caused by switching on and off the “DC recording” mode. *B*: superimposed five traces show short duration biphasic action potentials that were characteristic for the REM-ON neurons. *C*: a coronal pontine section stained for TH and neutral red shows the recording site of this neuron (white arrow). Note the distant location of TH-positive A7 neurons (black arrows) from the recording site, suggesting that the recorded neuron was not noradrenergic. CV, coefficient of variability; NREM, non-rapid-eye-movement; REM, rapid-eye-movement; TH, tyrosine hydroxylase.

Ten REM-active neurons were characterized by increased firing rate during REM sleep as compared with either NREM sleep or wakefulness. They were subdivided into two groups: low-frequency (LF, *n* = 7) and high-frequency (HF, *n* = 3) REM-active neurons (see Fig. 2, light and dark blue triangles, respectively). All LF and HF REM-active neurons were discharged with the “short” biphasic APs. The firing pattern of a representative LF neuron is shown in Fig. 7*A*. It had the “short” biphasic shape of AP (Fig. 7*B*). As shown in Fig. 7*C*, this neuron was recorded near the A7 nucleus, however, relatively far from TH-positive noradrenergic neurons. The average AP durations D3 and D4 in the seven LF REM-active neurons were significantly smaller than those of the eight A7 neurons [mean values: D1 = 0.238 ± 0.018 ms, D2 = 0.38 ± 0.021 ms, D3 = 0.546 ± 0.034 ms (*t* = 5.36, *P* < 0.001), D4 = 1.18 ± 0.97 ms (*t* = 3.68, *P* < 0.01)].

**Figure 8.**
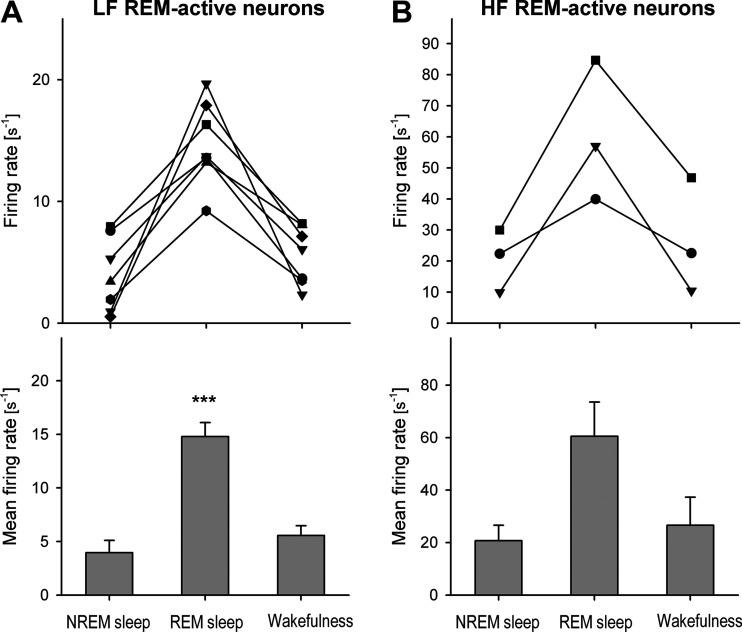
Group data for low- (*A*) and high- (*B*) frequency REM-ON neurons that were recorded during sleep and wakefulness in this study. *A*: mean firing rates of the REM-ON neurons were significantly different between all three behavioral states being minimal during NREM sleep and wakefulness, and maximal during REM sleep. ***P < 0.001, one-way RM ANOVA. HF, high-frequency; LF, low-frequency; NREM, non-rapid-eye-movement; REM, rapid-eye-movement.

The group data of individual and mean firing rates of the LF REM-active neurons that were recorded in this study are provided in Fig. 8*A*. On average, the LF REM-active cells had the following discharge rates: during NREM sleep (mean: 3.95 ± 1.2 s^−1^, range: 0.53–7.9 s^−1^; mean CV: 1.46 ± 0.3, range: 0.76–2.69; *n* = 7), REM sleep (mean: 14.8 ± 1.3 s^−1^, range: 9.23–19.7 s^−1^; mean CV: 1.78 ± 0.57, range: 0.531–4.44; *n* = 7), and wakefulness (mean: 5.57 ± 0.9 s^−1^, range: 2.33–8.18 s^−1^; mean CV: 1.47 ± 0.35, range: 0.579–2.92; *n* = 7). The increase in their activity during REM sleep was significant as compared with NREM sleep and wakefulness (*F*_2,6 = _26.7, *P* < 0.001, one-way RM ANOVA with *P* = 0.33 between NREM sleep and wakefulness, pairwise comparison by Holm–Sidak method).

**Figure 9.**
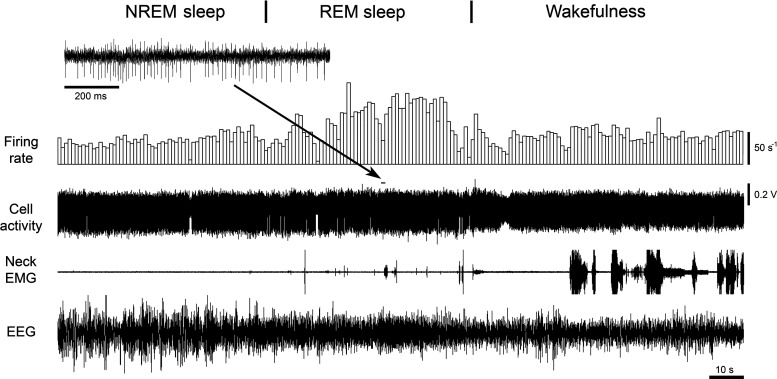
Example of spontaneous activity of a high-frequency REM-active neuron. This neuron discharged at higher frequency during REM sleep (average firing rate 84.6 s^−1^, CV 0.859) as compared with both NREM sleep (average firing rate 30 s^−1^, CV 0.356) and wakefulness (average firing rate 46.8 s^−1^, CV 0.42). CV, coefficient of variability; NREM, non-rapid-eye-movement; REM, rapid-eye-movement.

The individual and mean discharges of the HF REM-active neurons are shown in Fig. 8*B*. The HF REM-active neurons were discharged during NREM sleep at mean rate of 20.7 ± 5.9 s^−1^, range: 9.88–30 s^−1^, (mean CV: 1.09 ± 0.41, range: 0.356–1.77; *n* = 3); during REM sleep, at 60.5 ± 13 s^−1^, range: 39.9–84.6 s^−1^, (CV: 0.91 ± 0.066, range: 0.827–1.04; *n* = 3); and during wakefulness, at 26.6 ± 11 s^−1^, range: 10.4–46.8 s^−1^, (CV: 1.3 ± 0.44, range: 0.425–1.82; *n* = 3). The activity of a typical HF neuron during sleep and wakefulness is shown in Fig. 9.

### Other Neurons

#### REM/wake-active neurons.

**Figure 10.**
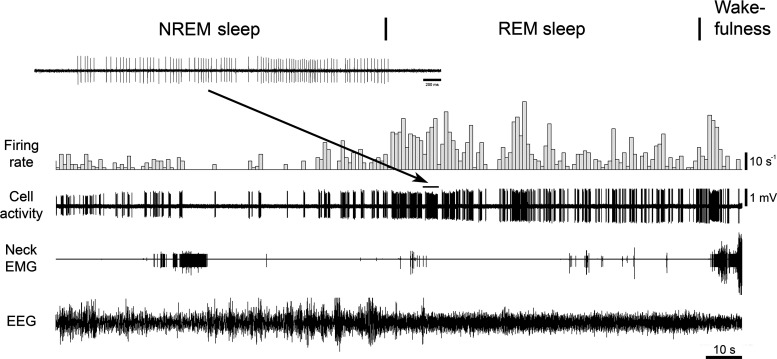
Example of the activity of a REM/wake-active neuron. This neuron discharged irregularly and at higher frequency during REM sleep (average firing rate 12 s^−1^, CV 2.69) and wakefulness (average firing rate 12.8 s^−1^, CV 3.5) as compared with NREM sleep (average firing rate 4.92 s^−1^, CV 1. 6). CV, coefficient of variability; NREM, non-rapid-eye-movement; REM, rapid-eye-movement.

**Figure 11.**
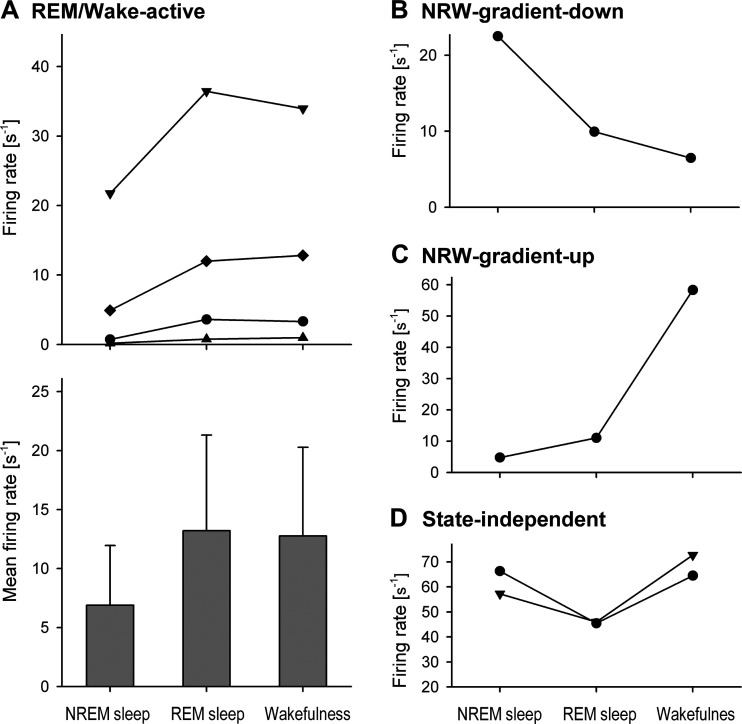
The firing rates of RW-active (*A*), NRW-gradient-down (*B*), NRW-gradient-up (*C*), and state-independent (*D*) neurons during sleep and wakefulness. NREM, non-rapid-eye-movement; NRW, NREM-REM-wake; REM, rapid-eye-movement; RW, REM/wake.

The pattern of activity of four REM/wake (RW)-active neurons was characterized by a low firing rate during NREM sleep and relatively high rates of discharge during both REM sleep and wakefulness. The activity of a typical RW-active neuron is shown in Fig. 10. One of these cells had the “long” triphasic AP, whereas the remaining two cells had the “short” biphasic APs. The RW-active cells discharged during NREM sleep at mean firing rate of 6.89 ± 5.1 s^−1^, range: 0.18–21.7 s^−1^ (mean CV: 1.39 ± 0.26, range: 0.83–2.0); during REM sleep, at mean firing rate of 13.2 ± 8.1 s^−1^, range: 0.8–36.4 s^−1^ (mean CV: 2.06 ± 0.46, range: 0.688–2.69); and during wakefulness, at mean firing rate of 12.8 ± 7.5 s^−1^, range: 0.974–33.9 s^−1^ (mean CV: 2.06 ± 0.53, range: 0.934–3.5). The individual and average discharges of the RW-active neurons are shown in Fig. 11*A*.

#### NREM-REM-wake-gradient neurons.

One NREM-REM-wake (NRW)-gradient neuron is characterized by a gradual decrease of its discharge from NREM sleep to REM sleep to wakefulness (NRW-gradient-down, Fig. 11*B*), whereas another neuron increased firing rate from NREM sleep to REM sleep to wakefulness (NRW-gradient-up, Fig. 11*C*). The NRW-gradient-up neuron had a “long” biphasic AP, whereas the NRW-gradient-down neuron had a “short” biphasic AP. Both NRW-gradient cells were fired more regularly during NREM sleep and wakefulness than during REM sleep. The NRW-gradient-up neuron discharged with mean frequency 4.78 s^−1^ (CV = 0.587) during NREM sleep, 11.0 s^−1^ (CV = 1.85) during REM sleep, and 58.3 s^−1^ (CV = 0.31) during wakefulness, while the mean firing rates of the NRW-gradient-down neuron were, respectively, 22.5 s^−1^ (CV = 0.419), 9. s^−1^ (CV = 3.6), and 6.5 s^−1^ (CV = 0.42).

#### State-independent neurons.

The remaining two cells tended to decrease their firing rate during REM sleep (Fig. 11*D*). However, the changes in their firing rates did not reach the 50% threshold and thus were classified as “state-independent.” Both neurons had “short” biphasic APs. One neuron discharged more regularly with the firing rate of 57.2 s^−1^ (CV = 0.211) during NREM sleep, 46.0 s^−1^ (CV = 0.686) during REM sleep, and 72.7 s^−1^ (CV = 0.278) during wakefulness, whereas the other cell discharged irregularly with the firing rate of 66.3 s^−1^ (CV = 0.939) during NREM sleep, 45.5 s^−1^ (CV = 2.94) during REM sleep, and 64.5 s^−1^ (CV = 1.2) during wakefulness.

#### Trigeminal motoneurons.

**Figure 12.**
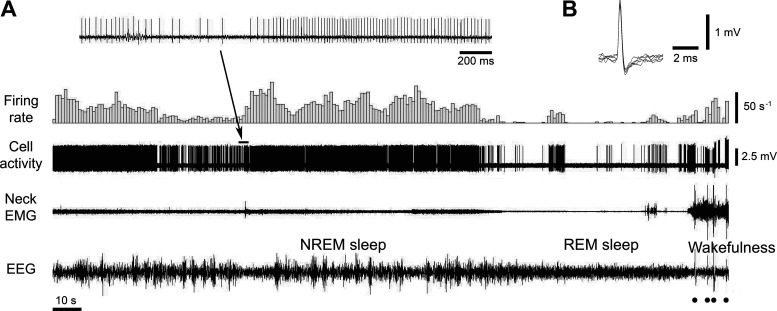
The activity of a trigeminal motoneuron that was recorded extracellularly during consecutive states of NREM sleep, REM sleep, and wakefulness. Typically, trigeminal motoneurons had relatively high amplitude “short” biphasic action potentials (*B*, superimposed five traces of action potential). They discharged with a varying frequency (up to 50 s^−1^) during NREM sleep (average firing rate of this motoneuron during NREM sleep was 19.4 s^−1^, CV 1.18). During REM sleep-induced muscle atonia, this motoneuron exhibited long periods of silence interrupted by sporadically appeared phasic discharges (average firing rate 2.65 s^−1^, CV 3.26). During wakefulness, its activity had mostly phasic pattern (average firing rate 21.2 s^−1^, CV 3.33). Filled circles indicate animal movement artifacts. The *inset* on *A* shows the motoneuron activity at expanded time scale. CV, coefficient of variability; NREM, non-rapid-eye-movement; REM, rapid-eye-movement.

The activity of trigeminal motoneurons served primarily as a reference for estimating the locations of A7 nuclei in each animal, therefore, their behavior was not systematically analyzed. Their discharges correlated with animal’s jaw movements during grooming and were characterized by relatively steady firing at a high frequency (range: 19.4–38.8 s^−1^; CV: 0.53–1.3; *n* = 3 cells) and the “short” biphasic APs. During REM sleep, the firing rate of trigeminal motoneurons was strongly decreased but was interrupted by short periods of phasic excitations (Fig. 12).

In addition, we noticed that the amplitude of the neuron’s APs often increased during REM sleep relative to NREM sleep and wakefulness, especially if relatively thin electrodes were used for recording. This was likely to result from upward micromovements of brain tissue relative to the tip of electrode during REM sleep, which could be explained by the increase in blood pressure due to the sympathetic activation during REM sleep (39–42). However, these micromovements did not affect the shape of APs of recorded neurons during transitions between wake, NREM and REM sleep.

## DISCUSSION

The main finding of this study is the characterization of the behavior of putative noradrenergic A7 neurons during sleep and wakefulness in head-restrained, undrugged, naturally sleeping rats. We have obtained the first direct evidence that noradrenergic neurons of the A7 group are silent or nearly silent during REM sleep, which is analogous to other pontine noradrenergic neurons that are located in LC (29), SubC (30), and A5 nuclei (27). However, unlike the typical behavior of pontine noradrenergic neurons, which discharge at a high rate during wakefulness and decrease their discharge during NREM sleep (29, 30), the frequency of putative A7 neuron firing rate during wakefulness and NREM sleep appeared to be similar. We also recorded the activity of REM-active neurons that were often located nearby the A7 nucleus.

We have recorded putative A7 noradrenergic neurons within the A7 nucleus, which was confirmed by distribution of TH-positive A7 neurons in the pontine lateral tegmentum. A limitation of his study is that the transmitter phenotype of the putative REM-off A7 neurons was not explicitly determined to be noradrenergic. However, the recorded A7 neurons had electrophysiological parameters that were characteristic of noradrenergic neurons: relatively long duration of action potentials (27, 43) and the regular, low-frequency discharge (27, 29, 30, 44). In addition, a decrease in c-Fos expression has been found in noradrenergic A7 neurons during REM sleep-like responses suggesting their REM-off behavior (32, 33).

In this study, all neurons that were recorded in the lateral pontine tegmentum had a wide spectrum of state-dependent patterns of activity: from REM-active to RW-active to NRW-gradient to state-independent neurons. The activity of most of the recorded neurons was modulated during wakefulness, NREM sleep, and REM sleep. This finding provides additional support for the important role of pontine neural circuitry in the regulation of the sleep-wake cycle (45–52). However, among the neurons recorded in this study, only putative A7 neurons had the REM-OFF pattern of activity. The REM-OFF behavior has been found in other pontine noradrenergic neurons located in LC, SubC, and A5 nuclei (27, 29–31). This finding is also in accord with cFos studies, in which a decrease in activity of noradrenergic A7 neurons during REM sleep in behaving rats (33) and during REM sleep-like state in anaesthetized rats (32) resulted in a marked decrease in cFos protein expression in A7 neurons. In addition, the recorded putative noradrenergic A7 neurons were insensitive to the motor behavior of animal during grooming unlike noradrenergic LC neurons that are sensitive to many peripheral sensory stimuli (53).

Spinally projecting A7 neurons are tonically inhibited by GABAergic mechanisms (54, 55). Major sources of GABAergic inputs to A7 neurons are the ventrolateral periaqueductal gray matter (56) and local GABAergic neurons (54). GABAergic neurons that are located in the oral pontine (PnO) and the caudal pontine (PnC) reticular nuclei express c-fos during REM sleep rebound period following selective deprivation (57–59). In our study, the duration of action potentials of REM-active neurons was similar to that of identified GABAergic neurons that were recorded within the dorsal raphe nucleus in rats (60). These data may suggest that at least some of the recorded REM-active neurons in this study could be GABAergic neurons, which may contribute to the inhibition of noradrenergic A7 neurons during REM sleep.

However, during NREM sleep and wakefulness, the behavior of the putative A7 neurons contrasted with that of the typical pontine noradrenergic neurons. Contrary to the stereotyped discharge pattern of the pontine noradrenergic neurons across the behavioral states (highly active during active wakefulness, less active during NREM sleep, and virtually silent during REM sleep (29, 30), we found that the average firing rate of putative A7 neurons appeared to be similar during both wakefulness and NREM sleep. This may suggest that noradrenergic A7 neurons have a limited contribution to the decrease of upper airway motoneurons activity during the transition from wakefulness to NREM sleep.

Most of the recorded cells in this study had “short” biphasic APs. We have found that the D3 duration of AP was closely correlated with D2 duration in these neurons. However, the D2 and D3 durations in the “long” AP neurons were not significantly correlated. In addition, we determined that the ratio of AP durations D3/D2 clearly separates the “long” AP from the “short” AP neurons at the ratio level 1.7. However, the number of recorded cells in this study was insufficient to generalize these findings to all pontine neurons. Therefore, more studies are needed to clarify if the D3/D2 ratio can be used to classify other neurons located in pons and in various brain regions.

We have found that the shape of AP among the recorded putative noradrenergic A7 neurons varied from the “long” biphasic to “LC-like” to triphasic. This difference in AP shapes may suggest different functions for A7 neurons such as contribution to sensorimotor control (61), pain modulation (54), or state-dependent modulation of upper airway activity (22, 23, 26). The remaining “long” AP cells were an RW-active neuron, an NRW-gradient-up neuron, and the two neurons, the state-dependent behavior of which were not classified. The RW-active neuron recorded in this study had a shape of AP that was similar to that of cholinergic neurons found within and around the laterodorsal and sublaterodorsal nuclei, some of which discharged at the highest rate during both wakefulness and REM sleep (62). Therefore, there is a possibility that this RW-active neuron could be cholinergic. This neuron was recorded in the lateral pons, where some cholinergic cells have been found in anatomical studies (48). The recorded NRW-gradient-up neuron in this study was located in the lateral parabrachial region and was mostly active during wakefulness. Perhaps, this NRW-gradient-up neuron can represent one of the wake-promoting neurons that were described in this region (63).

In this study, we used the shape of extracellularly recorded action potentials to help the classification of the recorded neurons, which is commonly performed in similar studies (27, 36, 62). The shape of AP of a neuron depends on both a unique composition of different types of ion channels that are characteristic for a given type of neuron and the timing of their opening and closing during AP generation. As the channel composition and timing are likely to be shared by neurons of certain neuronal groups, the shape of AP can be regarded as one of the features of neuronal phenotype. For example, the locus coeruleus noradrenergic neurons discharge with characteristic APs that are followed by a distinct “notch” (64).

However, it has been shown that the activation of metabotropic receptors, which alter the behavior of ionic channels, may affect the shape of neuronal AP. For instance, in in vitro studies, the inhibition of K^+^ currents by muscarinic agonists significantly changed AP shapes of recorded neurons (65, 66). The shape of AP may also depend on a neuronal firing rate (65). Therefore, we decided to verify whether the behavioral state of animal or the firing rate affected the shape of AP in the neurons recorded in the present study. We have found that the AP shape of each of the 27 fully recorded neurons did not change between wakefulness, NREM sleep, and REM sleep. However, some changes in the timing of AP were detected in two HF REM-active neurons at different discharge frequencies. In one neuron, the D2 and D3 durations of AP were 0.39 ms and 0.57 ms, respectively, at firing rate of 11 s^−1^ (D3/D2 = 1.45) but they increased to 0.43 ms and 0.66 ms, respectively, at firing rate of 103 s^−1^ (D3/D2 = 1.53). In another neuron, the D2 and D3 AP durations were 0.47 ms and 0.62 ms, at firing rate of 20 s^−1^ (D3/D2 = 1.33), 0.45 ms and 0.63 ms, at firing rate of 79 s^−1^ (D3/D2 = 1.39), and 0.42 ms and 0.63 ms, at firing rate of 209 s^−1^ (D3/D2 = 1.51). Thus, it appeared that there was a consistent trend for the increase in the D3/D2 ratio at elevated neuronal firing rates. However, despite the large changes in firing rate, the D3/D2 ratios of the two “short AP” neurons did not reach the threshold of the 1.7, which separated the “short” and “long” AP neurons in this study. Therefore, given the stability of the D3/D2 AP ratio, we believe that the shape of AP can be helpful to classify neuronal phenotypes despite the possibility that the AP shape may change under different behavioral conditions, which could not be verified in this study.

### Implications of Noradrenergic A7 Neurons in State-Dependent Control of Hypoglossal Motoneurons

Noradrenergic A7 neurons send axonal projections to the spinal cord where they contribute to gating sensorimotor and nociceptive neurotransmission of spinal motoneurons (54, 61, 67–69). In addition, retrograde neuroanatomical studies have provided evidence that the catecholaminergic neurons located in A1/C1, A5, SubC, and A7 brainstem nuclei strongly project to the hypoglossal nucleus with the largest afferent input originating from the A7 group (22, 23). In functional studies, the inhibition of A7 neurons by a local application of clonidine, an α_2_-adrenergic agonist, significantly decreased activity in hypoglossal nerve in anesthetized rats (26). However, identical local microinjections of clonidine into A5, LC, or SubC nuclei did not affect hypoglossal nerve activity in the same preparation (26–28). It was also found that A1/C1 cells moderately contribute to the maintenance of hypoglossal motoneurons in behaving mice (70). These data suggest that A7 neurons provide important noradrenergic excitatory drive to hypoglossal motoneurons.

The withdrawal of noradrenergic drive (17, 18, 20) and increase in cholinergic inhibition (19) are the two major neural mechanisms of REM sleep-related depression of hypoglossal motoneuron activity. As noradrenergic A7 neurons have been suggested to provide an important noradrenergic input to hypoglossal motoneurons (26), they may be involved in maintaining the patency of upper airway muscles and, thus, play a role in OSA pathophysiology (20). In this study, putative noradrenergic A7 neurons were mainly silent during REM sleep. This finding is in accordance with the proposed key role for these neurons in the withdrawal of noradrenergic drive from hypoglossal motoneurons during REM sleep. Therefore, modulation of the activity of noradrenergic A7 neurons or their afferents can be investigated in future translational research aiming to determine additional targets for pharmacological treatment of OSA. One possible treatment could aim to specifically increase the activity of A7 neurons. As activation of noradrenergic A7 neurons produces antinociception (71), one possible side effect of such treatment would be a loss or reduction of painful sensations.

## GRANTS

This study was supported by National Institutes of Health, Grants HL116845, AG065233, and HL133847.

## DISCLOSURES

No conflicts of interest, financial or otherwise, are declared by the authors.

## AUTHOR CONTRIBUTIONS

V.B.F. conceived and designed research; V.B.F. performed experiments; V.B.F. and I.R. analyzed data; V.B.F. interpreted results of experiments; V.B.F. prepared figures; V.B.F. drafted manuscript; V.B.F. and I.R. edited and revised manuscript; V.B.F. and I.R. approved final version of manuscript.
